# Long-Term Clinical Outcomes of Adults Hospitalized for COVID-19 Pneumonia

**DOI:** 10.3201/eid3106.241097

**Published:** 2025-06

**Authors:** Ivan O. Rosas, Alejandra Benitez, James A. McKinnell, Reena Shah, Michael Waters, Bradley D. Hunter, Robert Jeanfreau, Larry Tsai, Margaret Neighbors, Ben Trzaskoma, Rita de Cassia Castro, Fang Cai

**Affiliations:** Baylor College of Medicine, Houston, Texas, USA (I.O. Rosas); Genentech, South San Francisco, California, USA (A. Benitez, L. Tsai, M. Neighbors, B. Trzaskoma, R. de Cassia Castro, F. Cai); Torrance Memorial Medical Center, Torrance, California, USA (J.A. McKinnell); Aga Khan University Hospital, Nairobi, Kenya (R. Shah); Velocity Clinical Research, Chula Vista, California, USA (M. Waters); Intermountain Healthcare, Salt Lake City, Utah, USA (B.D. Hunter); Velocity Clinical Research, New Orleans, Louisiana, USA (R. Jeanfreau)

**Keywords:** COVID-19, respiratory infections, severe acute respiratory syndrome coronavirus 2, SARS-CoV-2, SARS, coronavirus disease, coronavirus, cognitive dysfunction, heart function tests, patient-reported outcome measures, post-acute COVID-19 syndrome, pulmonary function tests, viruses, zoonoses, Kenya, Peru, United States

## Abstract

We conducted a multicenter, observational, 12-month follow-up study to identify the extended health burden of severe COVID-19 pneumonia by characterizing long-term sequelae of acute infection in participants previously enrolled in clinical trials for severe COVID-19 pneumonia requiring hospitalization. Overall, 134 (77.5%) of 173 participants completed the study. At 12 months, 51 (29.5%) participants reported cough, 60 (34.7%) reported dyspnea, 56 (32.4%) had residual lung texture abnormalities on high-resolution computed tomography scans, 26 (15.0%) had impaired forced vital capacity, 52 (30.1%) had cognitive impairment, and 77 (44.5%) reported fatigue. Disease severity during acute infection and age were associated with persistent lung texture abnormalities; history of hypertension was associated with higher prevalence of fatigue and more frequent dyspnea and cough; and age and obesity were associated with long-term cognitive impairment. Our findings underscore the long-term health burden of severe COVID-19 pneumonia, reinforcing the importance of regular monitoring in older persons and those with underlying illnesses.

COVID-19, attributable to SARS-CoV-2, has been a considerable cause of acute lung injury, multiorgan failure, and death (*1*,*2*). With advances in vaccines and treatments, the incidence of severe outcomes has decreased; however, COVID-19 remains a public health concern (*3*). Some patients have reported prolonged symptoms after recovering from acute COVID-19, including fatigue, respiratory symptoms, cardiovascular symptoms, and abnormalities in cognitive function (*4*–*6*). Early clinical trials during the COVID-19 pandemic provided valuable data on novel therapeutic strategies aimed at reducing the illness and death associated with acute severe COVID-19 pneumonia; however, long-term outcomes are underreported (*7*–*11*).

Long-term respiratory, cardiovascular, and neurologic sequelae develop in some patients who have had severe COVID-19 pneumonia requiring invasive mechanical ventilation or intensive care unit admission (*4*–*6*). Pulmonary complications, such as a reduction in the diffusing capacity of the lungs for carbon monoxide (DLCO) and radiologic abnormalities, might persist for months (*12*). Mechanical ventilation for the treatment of severe COVID-19 pneumonia has been linked to the development of pulmonary fibrosis and worsening of underlying interstitial lung disease (ILD) (*13*). Cardiovascular complications, such as myocarditis, heart failure, and arrhythmias, have also been observed after COVID-19, possibly resulting from viral invasion of myocardial tissue, systemic inflammation, and microvascular damage (*14*). Patients with underlying cardiovascular disease are particularly at risk for severe outcomes and have prolonged recovery periods (*15*). Neurologic sequelae, including cognitive impairment, headaches, and peripheral neuropathy, have been reported in patients recovering from severe COVID-19 (*5*,*16*). Neurotropic properties of the virus and effects of systemic inflammation and hypoxia are thought to contribute to long-term neurologic issues (*17*). To identify the extended health burden of patients hospitalized with severe COVID-19 pneumonia, we conducted the Long-Term Outcomes Post-Acute COVID-19 (LOPAC) study, a multicenter, observational, 12-month follow-up study that explored long-term respiratory, cardiac, and neurocognitive sequelae occurring after acute COVID-19 pneumonia.

## Methods

### Participants and Assessments

Persons >18 years of age were eligible for enrollment in the LOPAC study if they had participated in a Genentech/Roche-sponsored parent study while hospitalized for COVID-19 pneumonia. The parent studies were clinical trials (registered with https://www.clinicaltrials.gov) as follows: EMPACTA (study no. NCT04372186), COVACTA (no. NCT04320615), MARIPOSA (no. NCT04363736), COVASTIL (no. NCT04386616), and REMDACTA (no. NCT04409262) (*7*–*11*). The parent studies recruited patients from a total of 15 countries. The LOPAC study was a follow-up to the parent studies that participants could opt into; no investigational drugs were evaluated in the LOPAC study. Participants provided written informed consent, and the study was conducted in accordance with the Declaration of Helsinki and Good Clinical Practice. The protocol was approved by institutional review boards or independent ethics committees at each study site.

**Figure 1:**
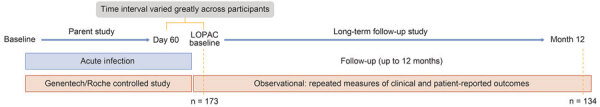
LOPAC study design to determine long-term clinical outcomes of adults hospitalized for COVID-19 pneumonia. Parent studies were sponsored by Genentech (https://www.gene.com) and Roche (https://www.roche.com). LOPAC, Long-Term Outcomes Post Acute COVID-19.

We defined baseline as the time of enrollment in the LOPAC study and conducted the baseline study visit after participants completed or discontinued their participation in the parent study; longitudinal study visits occurred every 3 months for 12 months (Figure 1). Key clinical measures were high-resolution computed tomography (HRCT), echocardiogram, and Montreal Cognitive Assessment (MoCA) performed at baseline and month 12; pulmonary function tests performed in a clinic every 3 months; and patient-reported outcomes recorded every 6 months. We assessed patient-reported outcomes by using the Short-Form 36 Question Health Survey version 2 (SF-36v2; Quality Metric, https://www.qualitymetric.com) and the Modified Living with Idiopathic Pulmonary Fibrosis (L-IPF-M) (*18*) symptoms questionnaires. We assessed healthcare resource utilization at each visit. We analyzed blood samples for SARS-CoV-2 antibodies every 6 months and for the *MUC5B* promoter variant rs35705950 (an allele associated with risk for idiopathic pulmonary fibrosis) at 1 visit. WorldCare Clinical, LLC (https://www.voiantclinical.com), conducted and managed independent centralized reads for HRCT scans and echocardiograms, which were also assessed by independent thoracic and cardiology readers. Participants had the option to consent to additional study assessments, including 6-minute walk tests (6MWTs), Borg dyspnea and fatigue scales, home spirometry, and daily monitoring of activity, heart rate, and sleep patterns through a wearable device (Appendix).

### Statistical Analyses

We reported categorical variables as counts and percentages. We reported percentages by using the total analysis population (to account for missing data at later visits) and continuous variables by using mean (SD) or median with range (minimum–maximum) or interquartile range. We considered data to be missing if obtained outside the assessment window, the assessment was performed but results were not evaluable, or the assessment was not performed at a study visit. Longitudinal analyses were descriptive and prespecified. We did not apply imputation methods.

To assess associations between lung texture abnormalities and selected pulmonary function tests (forced vital capacity [FVC], forced expiratory volume in 1 second [FEV_1_], and DLCO corrected for hemoglobin), we stratified summary statistics (mean [SD] of percent-predicted values) according to HRCT lung texture abnormality status at concurrent visits. We also stratified longitudinal FVC according to baseline FVC status; we defined abnormal FVC at baseline as <80% of the predicted value and normal as >80% of predicted value. We performed this analysis in the overall study population and in the subgroup of participants who attended all visits during baseline to month 12. We reported mean FVCs and 95% CIs.

We summarized L-IPF-M questionnaire responses by using mean patient-reported scores for each symptom domain: dyspnea (7 questions), coughing (5 questions), and fatigue (3 questions). Scores ranged from 0 to 3; the lowest score corresponded to no symptoms, and a higher score corresponded to greater symptom severity (*18*). We analyzed the proportion of participants with no symptoms (mean score 0) versus any symptom level (mean score 1–3). We summarized SF-36v2 scores by using the median for each of 8 domains: general health, mental health, physical functioning, bodily pain, role-emotional, role-physical, social functioning, and vitality; we defined normal as a score >50 points (range 0–100 points) for each domain (*19*).

We assessed associations between selected endpoints (lung outcomes, cognition, patient-reported outcomes) and selected medical history at LOPAC baseline to supplement the prespecified analyses. We stratified summary statistics according to the presence or absence of each selected medical condition and tested for significance by using a χ^2^ test, Student *t*-test, or Welch *t*-test. We also tested those endpoints for associations with age (>65 years) and disease severity during the COVID-19 acute phase (>4 on the ordinal scale at baseline of the parent study, requiring high-flow oxygen, invasive ventilation, or additional life support). Those analyses did not adjust for measured confounders, and we did not apply multiple testing adjustment. We used SAS version 9.4 (SAS Institute, Inc., https://www.sas.com) and R version 4.1.0 (The R Project for Statistical Computing, https://www.r-project.org) for analyses.

For descriptive purposes, we further summarized baseline characteristics and a subset of key study endpoints according to the treatment arm that participants were randomly assigned to in the parent studies. That summary resulted in 3 cohorts of LOPAC study participants: those who received tocilizumab as a study drug (including participants randomly assigned to receive tocilizumab and remdesivir), those who received a study drug other than tocilizumab (i.e., remdesivir, astegolimab, or efmarodocokin alfa), and those who received placebo. However, the LOPAC study was not designed to compare the long-term efficacy of tocilizumab versus other study drugs or placebo; therefore, we made no formal comparisons.

## Results

**Figure 2:**
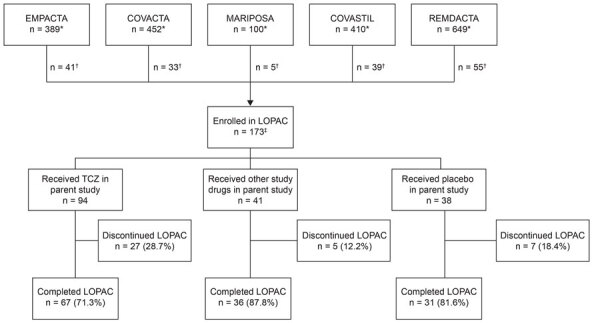
Disposition of LOPAC study participants in study of long-term clinical outcomes of adults hospitalized for COVID-19 pneumonia. The parent studies were clinical trials (registered with https://www.clinicaltrials.gov) as follows: EMPACTA (study no. NCT04372186), COVACTA (no. NCT04320615), MARIPOSA (no. NCT04363736), COVASTIL (no. NCT04386616), and REMDACTA (no. NCT04409262). Participants might have received remdesivir or another antiviral drug as part of the standard of care in the EMPACTA, COVACTA, and MARIPOSA studies. Participants who were randomly assigned to receive placebo, astegolimab, or efmarodocokin alfa might have also received TCZ or remdesivir or both as part of the standard of care in the COVASTIL study. Participants randomly assigned to receive TCZ or placebo in the REMDACTA study also received remdesivir. *Number of participants in each parent study; †number of participants from each parent study who participated in the LOPAC study; ‡1 participant initially enrolled in the LOPAC study did not receive treatment in the parent study and was not included in any analyses for the parent or LOPAC study. LOPAC, Long-Term Outcomes Post Acute COVID-19 study; TCZ, tocilizumab.

We enrolled 173 participants from 29 centers in the United States, Kenya, and Peru who had been previously hospitalized for COVID-19 pneumonia and had participated in 1 of the 5 parent studies. Of the 173 participants, 134 (77.5%) completed and 39 (22.5%) did not complete the LOPAC study (Figure 2). Of the 173 enrolled participants, 94 had been randomly assigned to receive tocilizumab, 41 had been assigned to receive another study drug, and 38 had been assigned to receive placebo in the parent study. Of the 94 participants who had been randomly assigned to receive tocilizumab in the parent study, 58 had been assigned to receive tocilizumab only, and 36 had been assigned to receive tocilizumab plus remdesivir. Overall, the reasons for early discontinuation from the LOPAC study were participant withdrawal (n = 18), loss to follow-up (n = 17), physician decision (n = 2), death (n = 1), and other (n = 1). Early discontinuation occurred in 28.7% (27/94) of participants randomly assigned to receive tocilizumab, 12.2% (5/41) of participants randomly assigned to receive another study drug, and 18.4% (7/38) randomly assigned to receive placebo (Figure 2).

We recorded demographics and clinical characteristics of the study participants (Appendix Table 1). The mean age of participants was 56.3 + 11.9 years. The most common underlying illnesses were hypertension (63.0%), obesity (46.8%), hyperlipidemia (43.9%), diabetes (35.3%), and asthma (11.0%). At the time of enrollment in the parent studies, 158 (91.3%) participants were not on mechanical ventilation, whereas 15 (8.7%) required mechanical ventilation or extracorporeal membrane oxygenation. At the completion of the parent study, almost all (97.4%) participants were discharged or ready for discharge from the hospital. The median duration of hospital stay during the parent study was 11 (range 3–74) days, and the median time from finishing the parent study to enrolling in LOPAC was 155 (range −1 to 338) days. At the time of enrollment in the LOPAC study, 2 (1.2%) participants required supplemental oxygen.

### HRCT Lung Texture Abnormalities and Impaired Pulmonary Function

At least 1 lung texture abnormality on HRCT scan was evident in 91 (52.6%) participants at baseline (i.e., at enrollment into the LOPAC study) and 56 (32.4%) participants at month 12 (Table 1). Ground-glass opacification was the most frequently observed abnormality, found in 86 (49.7%) participants at baseline and 51 (29.5%) participants at month 12. Other lung texture abnormalities were reticular pattern in 15 (8.7%) participants at baseline and 10 (5.8%) at month 12, bronchiectasis in 12 (6.9%) participants at baseline and 7 (4.0%) at month 12, and hyperlucency in 2 (1.2%) participants at both time points. No participants had honeycombing at baseline or month 12.

**Table 1:** Proportion of participants with lung texture abnormalities on HRCT scan in LOPAC study of long-term clinical outcomes of adults hospitalized for COVID-19 pneumonia*

HRCT characteristics	No. (%) participants	
	LOPAC study, baseline	LOPAC study, month 12
Participants with >1 lung texture abnormality	91 (52.6)	56 (32.4)
Ground-glass opacification	86 (49.7)	51 (29.5)
Reticular pattern	15 (8.7)	10 (5.8)
Bronchiectasis	12 (6.9)	7 (4.0)
Hyperlucency†	2 (1.2)	2 (1.2)
Honeycombing	0	0
Participants with no lung texture abnormality‡	71 (41.0)	62 (35.8)
Participants with missing data	11 (6.4)	55 (31.8)

*Total number of participants enrolled in the LOPAC study was 173. HRCT, high-resolution computed tomography; LOPAC, Long-Term Outcomes Post Acute COVID-19. †Radiographic evidence of hyperlucency was assessed as unknown in 1 participant’s baseline and month 12 HRCT images. This participant was considered to have radiographic evidence of hyperlucency at baseline and month 12. ‡Participants were considered to have no lung texture abnormality if there was no radiologic evidence of ground-glass opacification, reticular pattern, bronchiectasis, hyperlucency, or honeycombing on HRCT scan.

**Figure 3:**
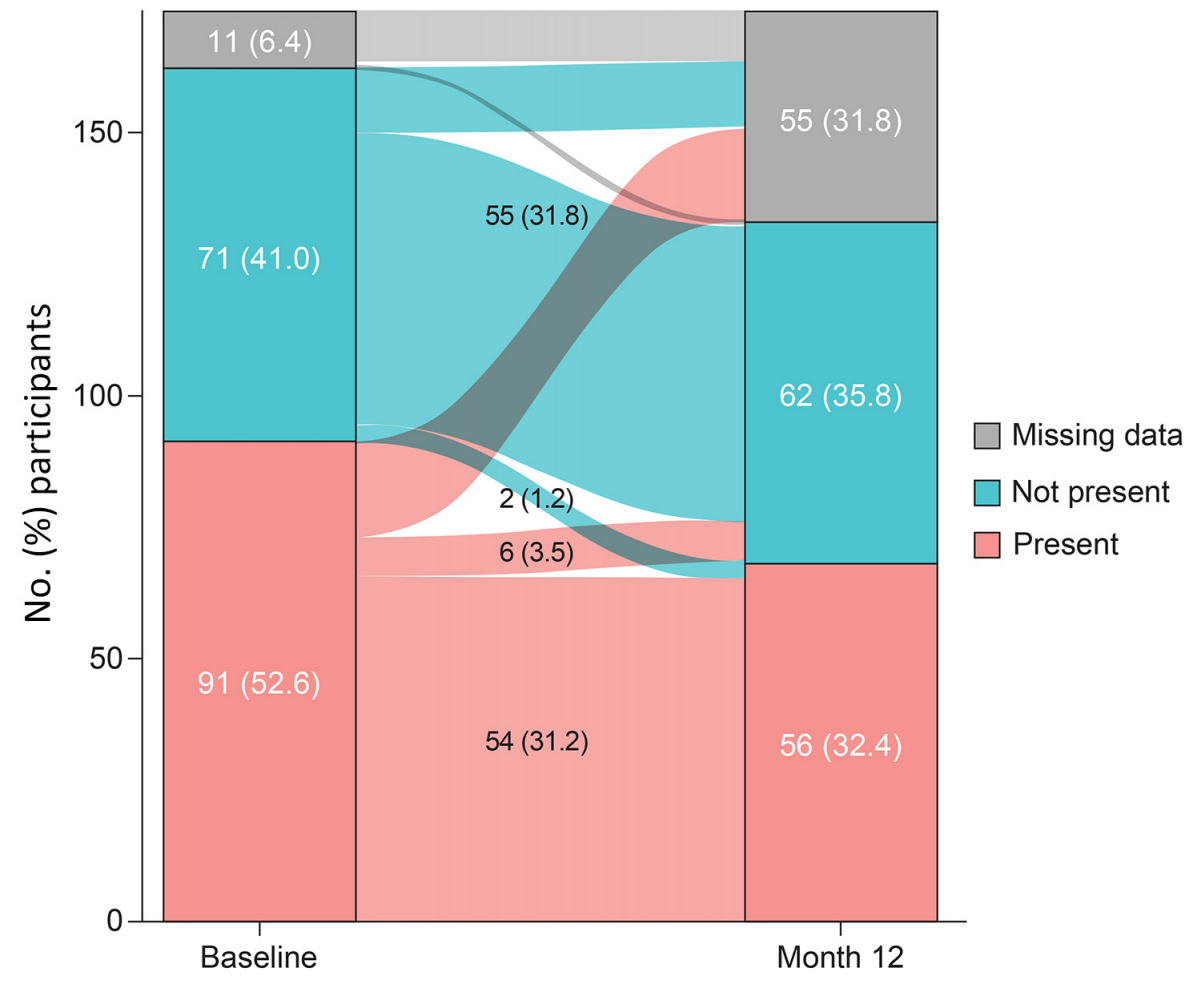
Numbers of participants with >1 lung texture abnormality at baseline and at month 12 in study of long-term clinical outcomes of adults hospitalized for COVID-19 pneumonia. Data are shown for a total of 173 participants enrolled in the Long-Term Outcomes Post Acute COVID-19 study. Colors indicate whether the lung texture abnormality was present or absent. Baseline and month 12 columns indicate the no. (%) participants who had >1 lung texture abnormality at that time point. Numbers between those columns indicate lung abnormalities that persisted, developed, or resolved by month 12.

Of the 173 participants, 54 (31.2%) had >1 lung abnormality at baseline that was still present at month 12, and 55 (31.8%) had no abnormality at either time point (Figure 3). Six (3.5%) participants who had >1 lung texture abnormality at baseline showed resolution at month 12, whereas ≥1 lung abnormality had developed at the 12-month follow-up in 2 (1.2%) participants who had no abnormalities at baseline.

At baseline, the mean FVC was 84.5% + 16.6%, and the mean FEV_1_ was 87.8% + 19.9%. FVC and FEV_1_ remained stable across the 12-month follow-up period; the mean change from baseline to month 12 was 2.8% + 9.1% for FVC and 1.7% + 10.3% for FEV_1_. Among 90 participants with DLCO measurements available at baseline, the mean was 83.3% + 21.1%; DLCO remained stable with a mean change from baseline of 1.8% + 13.8% at month 12.

**Figure 4:**
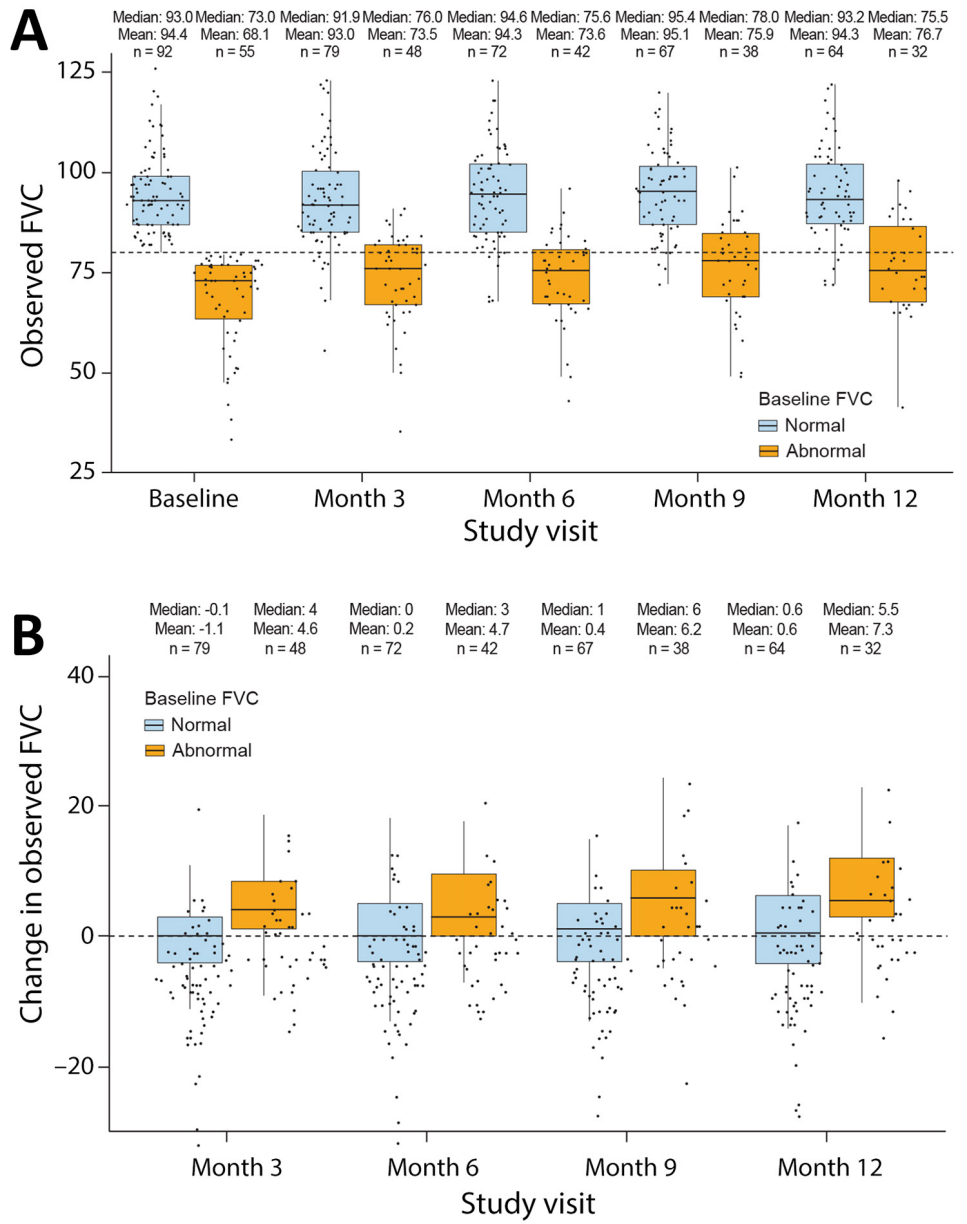
Box plots of longitudinal FVC profiles from the Long-Term Outcomes Post Acute COVID-19 study of adults previously hospitalized for COVID-19 pneumonia. A) Observed percent-predicted FVC at indicated study visits. B) Change in percent-predicted FVC at different study visits according to the baseline FVC. Dashed horizonal lines indicate 80% predicted FVC (A) and no change in percent-predicted FVC (B). Horizontal lines within boxes indicate medians; box tops and bottoms indicate upper (third) and lower (first) quartiles; error bars (whiskers) indicate 1.5 times interquartile range.. Dots indicate data points; data points beyond the end of the whiskers are considered outliers. Numbers above the bars indicate mean and median percent-predicted FVC and total number of participants in category. Abnormal is defined as <80% and normal ≥80% predicted FVC. FVC, forced vital capacity.

For most participants who had normal FVC at baseline, FVC appeared to remain stable, and the mean change from baseline appeared to be minimal during the 12-month follow-up (Figure 4, panels A, B). Many participants with abnormal FVC at baseline had an abnormal FVC at the 12-month visit; however, the mean change from baseline to 12 months improved overall. In participants with abnormal baseline FVC who completed the month 12 visit, the mean change from baseline in FVC was 7.3% (Figure 4, panel B). Longitudinal 95% CI of the mean FVC and FVC change from baseline (stratified by baseline FVC) showed consistent results (Appendix Figures 1, 2). We observed an inverse association between DLCO and lung texture abnormalities; we did not observe associations between lung texture abnormalities and FVC or FEV_1_ (Appendix Table 2).

Participants who had more severe acute infection (ordinal scale score >4) at enrollment in the parent study were more likely to have >1 lung texture abnormality on HRCT scan (62/101 [61.4%]) than those who had an ordinal scale score of <4 (29/72 [40.3%]); that finding was still apparent at month 12 for participants with scores >4 (41/101 [40.6%]) versus those with scores <4 (15/72 [20.8%]) (Appendix Figure 3, panel A). Participants >65 years of age were more likely to have lung texture abnormalities through month 12 (Appendix Figure 3, panel B). Participants with a history of hypertension tended to have lower median FVC at LOPAC baseline; however, by month 12, no apparent difference in median FVC between participants with or without a history of hypertension was observed (Appendix Figure 4). No association was detected between *MUC5B* promoter allele status and the presence of >1 lung texture abnormality or FVC abnormality at baseline or month 12 (Appendix Table 3). Some numeric differences were observed in pulmonary function and lung texture abnormalities between the 3 treatment cohorts (participants who received tocilizumab as a study drug, those who received a study drug other than tocilizumab, and those who received placebo in the parent studies) (Appendix Tables 4, 5).

### Cardiac Function

Overall, cardiac function was unremarkable at baseline and did not deteriorate by month 12. Mean left ventricular ejection fraction was 61.8% + 8.0% at baseline for 156 participants and 63.1% + 8.6% at month 12 for 115 participants with evaluable results (reference range 52%–72% for male patients, 54%–74% for female patients). Mean pulmonary artery pressure was 13.7 + 4.5 mm Hg at baseline for 43 participants and 12.5 + 2.9 mm Hg at month 12 for 36 participants with evaluable results (reference range <25 mm Hg). At baseline, 142 (82.0%) participants had unimpaired right ventricular systolic function (RVSF) (evaluated either by endocardial tracing or by medical assessment), 10 (5.8%) had mild impairment, 1 (0.6%) had severe impairment, and 20 (11.6%) had missing results (Table 2). At month 12, a total of 98 (56.6%) participants had unimpaired RVSF, 13 (7.5%) had mild impairment, and 62 (35.8%) had missing results. The participant with severe RVSF impairment at baseline was lost to follow-up at month 12.

**Table 2:** RVSF at baseline and month 12 of LOPAC study of long-term clinical outcomes of adults hospitalized for COVID-19 pneumonia*

RVSF characteristics	No. participants (%)	
	LOPAC study, baseline	LOPAC study, month 12
Evaluable by endocardial tracing†	74 (42.8)	58 (33.5)
Normal‡	71 (41.0)	56 (32.4)
Mildly impaired	2 (1.2)	2 (1.2)
Severely impaired	1 (0.6)	0§
Not evaluable by endocardial tracing, medical assessment only¶	79 (45.7)	53 (30.6)
Normal‡	71 (41.0)	42 (24.3)
Mildly impaired	8 (4.6)	11 (6.4)
Severely impaired	0	0
Total evaluable	153 (88.4)	111 (64.2)
Normal‡	142 (82.0)	98 (56.6)
Mildly impaired	10 (5.8)	13 (7.5)
Severely impaired	1 (0.6)	0§
Participants with missing data	20 (11.6)	62 (35.8)
Assessment done, not evaluable by endocardial tracing or medical assessment	5 (2.9)	6 (3.5)
Assessment done, not in window	2 (1.2)	15 (8.7)
Assessment not done	13 (7.5)	41 (23.7)

*Total number of participants enrolled in the LOPAC study was 173. LOPAC, Long-Term Outcomes Post Acute COVID-19; RVSF, right ventricular systolic function. †RVSF was measured by echocardiogram endocardial tracing, and the impairment level was assessed qualitatively by medical personnel. ‡Right ventricular fractional area change of >35% was classified as normal RVSF. Assessment of RVSF for 8 observations with right ventricular fractional area of <35% was obtained by medical personnel in a post hoc assessment. §Participant who had severely impaired RVSF at baseline was lost to follow-up at month 12. ¶RVSF could not be measured by endocardial tracing and was assessed only qualitatively by medical personnel.

### Cognitive Impairment

At baseline, the median MoCA score was 25 (range 9–30), and 87 (50.3%) of 173 participants exhibited cognitive impairment, indicated by MoCA scores below the threshold of 26. At month 12, a total of 52 (30.1%) participants had a MoCA score of <26, and 60 (34.7%) participants were missing from the assessment. For 113 participants who had MoCA scores assessed at both baseline and month 12, the median score increased by 1 point (range −9 to 10) by month 12.

Overall, 49 (28.3%) of 173 participants were >65 years of age. That age group had a lower mean change (−0.3) in MoCA scores from baseline to month 12 than participants who were <65 years of age (1.1) (p = 0.043). An association was observed between a change in MoCA score and obesity (p = 0.007) (Appendix Figure 5). Mean and median ages were similar between obese and nonobese participants. We also calculated median MoCA scores for the 3 treatment cohorts from the parent studies (Appendix Table 6).

### Patient-Reported Outcomes and Respiratory Symptoms

**Figure 5:**
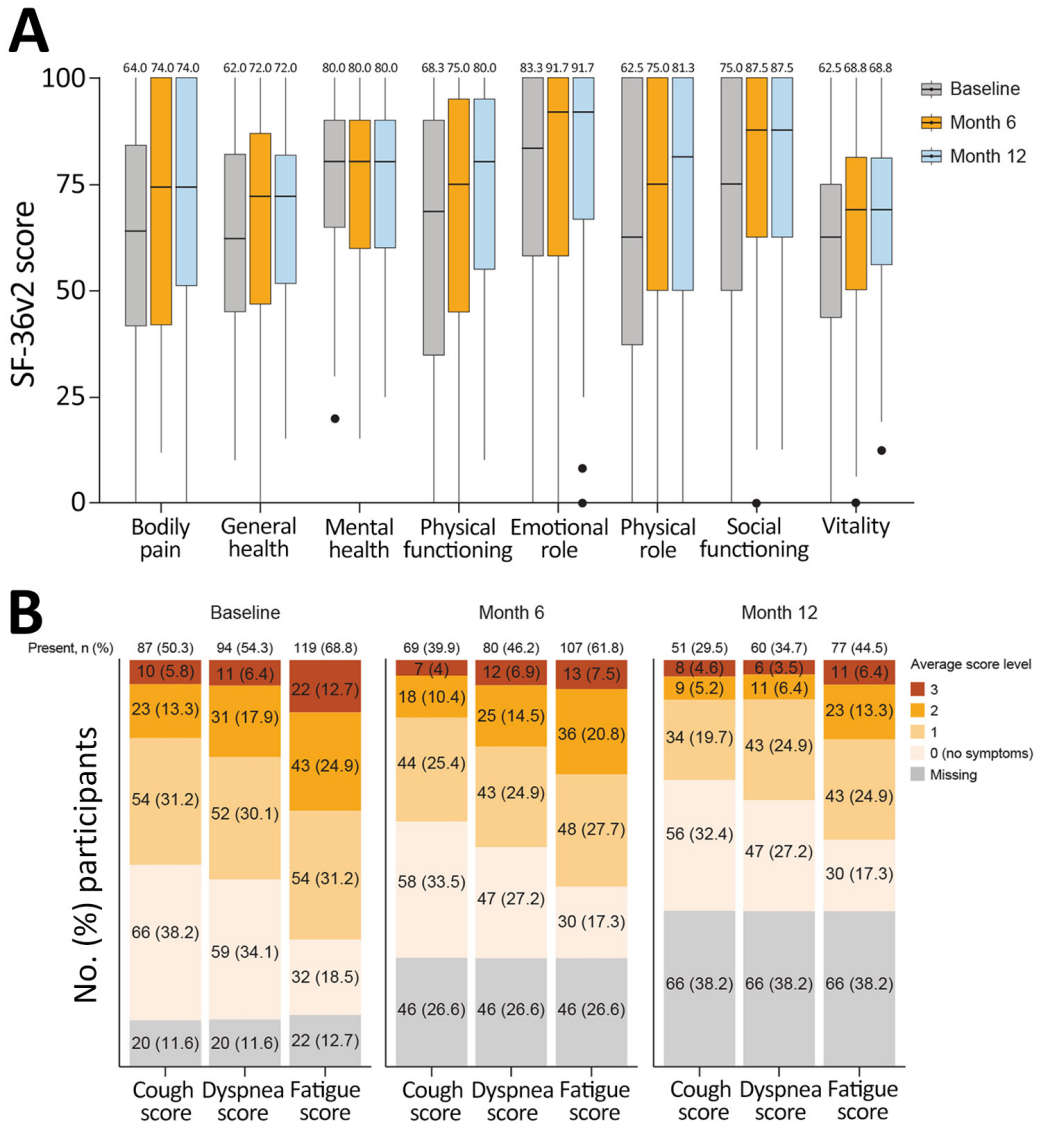
Longitudinal profiles of patient-reported outcomes from the Long-Term Outcomes Post Acute COVID-19 study of adults previously hospitalized for COVID-19 pneumonia. A) Box plots indicate quality of life assessed by SF-36v2 (Quality Metric, https://www.qualitymetric.com). SF-36v2 scores (range 0–100 points) were summarized by using the median for each of the 8 indicated domains. Normal was defined as a score of >50 points for each domain. Median participant scores within each domain are indicated at the top. Horizontal lines within boxes indicate medians; box tops and bottoms indicate upper (third) and lower (first) quartiles; error bars (whiskers) indicate 1.5 times interquartile range. Closed circles indicate outliers. B) Symptoms of study participants assessed by the Modified Living With Pulmonary Fibrosis Assessment Tool. Data are shown as no. (%) participants experiencing each category of symptoms according to 173 enrolled participants. Baseline refers to study baseline (time of enrollment). Questionnaire responses were summarized by using mean patient-reported scores for each symptom domain: dyspnea (7 questions), coughing (5 questions), and fatigue (3 questions). Scores ranged from 0 to 3; the lowest score corresponds to no symptoms and the higher score corresponds to greater symptom severity. SF-36v2, Short Form 36 Question Health Survey version 2.

At baseline, the median SF-36v2 questionnaire scores for all 8 domains (general health, mental health, physical functioning, bodily pain, role-emotional, role-physical, social functioning, and vitality) were >50 points (score range 0–100 points) for each domain; a score of 50 is the considered the threshold for a normal SF-36v2 score (*19*) (Figure 5, panel A). Lower median scores were reported for the general health (62.0), role-physical (62.5), vitality (62.5), bodily pain (64.0), and physical functioning (68.3) relative to social functioning (75.0), mental health (80.0), and role-emotional (83.3) domains. For 117 participants who completed the SF-36v2 questionnaire at month 12, median scores consistently increased from baseline to month 12 across all 8 domains. We also calculated mean and median scores for vitality, which denotes the absence of fatigue, for the 3 treatment cohorts from the parent studies (Appendix Table 7).

At baseline, 119 (68.8%) of 173 participants reported fatigue (lack of energy), 87 (50.3%) reported coughing, and 94 (54.3%) reported shortness of breath (dyspnea) on the L-IPF-M questionnaire (Figure 5, panel B). At month 12, a subgroup of participants still reported fatigue (44.5%), coughing (29.5%), or dyspnea (34.7%). Dyspnea, cough, and fatigue symptoms were more likely to be reported for participants who had hypertension at LOPAC baseline than for those who did not (Appendix Figure 6). Among participants who remained in the study at month 12, cough and fatigue were still frequently reported in those with hypertension compared with those without hypertension. We prepared descriptive summaries of healthcare resource utilization, weekly average walking duration, heart rate, and sleep duration (Appendix Table 8; Appendix Figure 7); further analysis is warranted to identify potential trends in those data.

## Discussion

We investigated long-term outcomes in the 12-month LOPAC follow-up study conducted with 173 participants who had been hospitalized for COVID-19 pneumonia and had participated in a Genentech/Roche-sponsored intervention parent trial. At baseline in the LOPAC study, >50% of participants had abnormalities on HRCT scans and ≈33% had lung function impairment. The high prevalence of lung abnormalities at baseline, measured at a median of 155 days after participants completed the parent study, aligns with postacute COVID-19 syndrome and meets the definition for long COVID (*20*–*22*).

During the 12-month follow-up, a subgroup of participants continued to exhibit clinical and radiologic abnormalities indicative of long-term COVID-19 sequelae, including abnormal lung texture, impaired pulmonary function, and symptoms of fatigue, coughing, or shortness of breath. The primary lung texture abnormality reported in participants in this study was ground-glass opacification, a nonspecific finding associated with increased lung density and preservation of the bronchopulmonary vasculature (*23*,*24*). Ground-glass opacities might be observed in acute and chronic pulmonary disease and might represent interstitial or alveolar involvement (or both) (*23*,*25*). Ground-glass opacification in COVID-19 characteristically has a bilateral, peripheral distribution, often in the posterior lung segments (*25*). The slow resolution of ground-glass opacities might suggest the development of parenchymal lung disease with ILD features, triggered or exacerbated by COVID-19 (*26*). Those findings contrast with previous reports that suggested ILD-like conditions resolve within a year after SARS-CoV-2 infection (*27*) and necessitate further investigation to prevent parenchymal disease progression and chronicity.

During the acute phase of COVID-19 pneumonia, an ordinal scale score >4 was recorded for 101 (58.4%) of 173 LOPAC study participants. That subgroup was at higher risk for prolonged lung texture abnormalities, consistent with previous reports of increased risk for parenchymal lung changes and fibrosis in patients with severe acute COVID-19 pneumonia (*28*) who required mechanical ventilation (*29*–*32*). Fibrotic changes after infection with severe acute respiratory syndrome (*33*) and Middle East respiratory syndrome (*34*) coronaviruses have also been reported.

In this study, participants who had not required mechanical ventilation or extracorporeal membrane oxygenation during acute infection experienced prolonged abnormalities in lung function and texture during follow-up. Older age, a known risk factor for SARS-CoV-2 susceptibility (*35*) and poor COVID-19 clinical outcomes (*36*), was associated with persistent lung abnormalities and cognitive impairment. Older age might compromise the immune system and hinder recovery from COVID-19. Our findings support the hypothesis that age-related factors, such as immune response changes and reduced regenerative capacity, might affect acute and long-term clinical outcomes (*37*).

The prevalence of respiratory and fatigue symptoms was higher in participants with hypertension than in those without hypertension. Hypertension, a key component of metabolic syndrome, is a well-recognized risk factor for severe COVID-19, likely through dysregulation of the renin-angiotensin-aldosterone system, altered immune responses, gastrointestinal disturbances, and increased inflammation; all of which might have prolonged effects on organs, including lungs, heart, and brain, and contribute to persistent symptoms (*38*). Obese participants exhibited persistent cognitive impairment compared with nonobese participants. The association between obesity and cognitive issues suggests potential links between metabolic factors, chronic inflammation, and neurologic consequences of COVID-19 (*17*). Although underlying conditions might influence some aspects of postacute COVID-19 syndrome, their effects vary depending on the specific examined symptom or health aspect. Further research will be required to clarify those associations.

The main contribution of this prospective study is the longitudinal assessment of lung texture; pulmonary, cardiac, and cognitive function; quality of life; and serology that captures detailed and standardized individual-level data. We also investigated baseline clinical characteristics and their associations with key clinical endpoints. However, the first limitation of our study is that we enrolled a small group of participants from each parent study to characterize long-term sequelae of severe COVID-19; we did not design the study to evaluate treatment effects during initial infection on long-term outcomes. In addition to the study drug received in the parent study, all participants received standard-of-care treatment during the acute phase of infection. As the standard-of-care treatments evolved during the COVID-19 pandemic, participants might have received tocilizumab, remdesivir, or both as part of standard care (*7*–*11*). Second, limited information was available on participants before hospitalization for acute COVID-19, and some participants might have been living with undetected underlying conditions. Third, evaluations were limited by baseline imbalances, the 22.5% discontinuation rate, and missing assessments, which are common in long-term follow-up studies. Although some potential confounding factors were identified, we did not adjust for them in this exploratory study. Further investigation adjusting for confounders will be necessary to confirm those findings and further delineate the long-term effects of COVID-19.

In conclusion, a subgroup of participants in this study exhibited persistent lung texture abnormalities, impaired pulmonary function, cognitive impairment, or reported fatigue or respiratory symptoms during the 12-month follow-up period, demonstrating a substantial long-term health burden for persons who have severe COVID-19 pneumonia. The severity of acute infection, age, and certain underlying conditions might influence the long-term sequelae of COVID-19. Our findings underscore the extended health effects of COVID-19 beyond its acute phase and reinforce the importance of regular monitoring of patients with severe COVID-19, particularly in older patients and those with underlying health conditions.

Appendix Additional information for long-term clinical outcomes of adults hospitalized for COVID-19 pneumonia.
