# Supplementary material for: Long-Term Clinical Outcomes of Adults Hospitalized for COVID-19 Pneumonia
Source: Emerg Infect Dis. 2025 Jun;31(6):1158–68. doi: 10.3201/eid3106.241097 (PMC12123926; doi:10.3201/eid3106.241097)
Supplement: Appendix — Additional information for long-term clinical outcomes of adults hospitalized for COVID-19 pneumonia. [file 24-1097-Techapp-s1.pdf]

Article DOI: https://doi.org/10.3201/eid3106.241097

EID cannot ensure accessibility for supplementary materials supplied by authors. Readers who have difficulty accessing supplementary content should contact the authors for assistance.

Long-Term Clinical Outcomes of Adults Hospitalized for COVID-19 Pneumonia

Appendix

Additional Methods

Status of Idiopathic Pulmonary Fibrosis Risk Allele rs35705950 of *MUC5B*

The associations between the idiopathic pulmonary fibrosis risk allele rs35705950 in the *MUC5B* promoter and lung texture abnormalities or lung function impairment (forced vital capacity [FVC] <80% of the predicted value) were assessed at baseline and month 12. Fisher exact tests were used to evaluate those associations.

SARS-CoV-2 Antibodies

SARS-CoV-2 antibodies in samples collected at baseline, month 6, and month 12 were measured by using Elecsys (Roche Diagnostics, https://www.roche.com).

In-Home Spirometry Monitoring

In addition to in-clinic pulmonary function assessments, study participants were invited to conduct weekly home monitoring of FVC/forced expiratory volume in 1 second (FEV_1) throughout the study by using a handheld spirometry device.

Continuous Monitoring of Activity, Sleep, and Heart Rate

Participants were invited to use an optional wearable device, such as an Apple Watch, to capture daily activity, sleep patterns, and heart rate for the duration of the study.

Additional Results

Mean FVC and Mean Change from Baseline in FVC According to FVC Status at Baseline of the Long-Term Outcomes Post-Acute COVID-19 (LOPAC) Study

For participants with a normal FVC at baseline, the mean FVC did not vary greatly from baseline to month 12 (Appendix Figure 1, panel A). Participants with an abnormal FVC at baseline had an apparent increase in mean FVC from baseline to month 12; however, FVC remained abnormal at month 12 in most of those participants. Participants with a normal FVC at baseline had little change from baseline in mean FVC at each follow-up visit to month 12 (Appendix Figure 1, panel B). Participants with an abnormal FVC at baseline showed an apparent increase in mean change in FVC through month 12. Among participants who completed FVC assessments at all visits from baseline through month 12, the longitudinal FVC profiles were similar to those in the overall study population (Appendix Figure 2).

Status of *MUC5B* Promoter Risk Allele

No association was detected between the status of the *MUC5B* promoter risk allele, rs35705950, and the presence of $\geq$1 lung texture abnormality at baseline ($p = 0.474$) or month 12 ($p>0.999$), nor was there an association with impaired FVC at baseline ($p = 0.227$) or month 12 ($p = 0.727$) (Appendix Table 3).

Healthcare Resource Utilization and SARS-CoV-2 Antibody Testing

Among the 173 enrolled participants, 61 (35.3%) reported using telephone health advice, 57 (32.9%) reported attendance at the hospital as an outpatient, 43 (24.9%) reported an emergency department visit, 35 (20.2%) reported physical therapy utilization, and 70 (40.5%) reported other healthcare resource utilization. Utilization was participant-reported; specific details of the underlying conditions leading to resource utilization were not collected (Appendix Table 8). Of 163 participants tested for SARS-CoV-2 antibodies, 158 had detectable SARS-CoV-2 spike protein–specific antibodies at baseline, and all participants tested at months 6 and 12 had detectable levels.

Six-Minute Walk Test

Six-minute walk test (6MWT) results were available for 116 participants at LOPAC baseline and 84 participants at month 12. Overall, the mean 6MWT distance did not worsen during the 12-month follow-up period. The mean $\pm$ SD distance was 359.9 $\pm$ 118.5 m at baseline

and 363.0 ± 105.4 m at month 12. The mean oxygen saturation levels were 96.7% ± 2.1% before and 96.2% ± 3.6% after the 6MWT at LOPAC baseline; the mean change was −0.5% ± 3.2%. At month 12, the mean oxygen saturation levels were 96.6% ± 1.7% before and 95.9% ± 2.9% after the 6MWT; the mean change was −0.7% ± 3.0%. The mean heart rates at LOPAC baseline were 76.7 ± 13.6 beats/min before and 88.7 ± 19.1 beats/min after the 6MWT; the mean change was 11.9 ± 14.0 beats/min. At month 12, the mean heart rates were 77.5 ± 15.9 beats/min before and 88.2 ± 18.6 beats/min after the 6MWT; the mean change was 10.8 ± 19.1 beats/min.

Borg scales

Borg scales range from 0 to 10; a higher rating indicates greater effort or difficulty in breathing or greater levels of fatigue. Mean Borg scale dyspnea scores at LOPAC baseline were 0.4 ± 0.9 before and 1.6 ± 2.1 after the 6MWT; the mean change was 1.2 ± 1.9. At month 12, the mean Borg scale dyspnea scores were 0.6 ± 1.2 before and 1.7 ± 2.1 after the 6MWT; the mean change was 1.1 ± 1.4. The mean Borg scale fatigue scores at LOPAC baseline were 0.6 ± 1.2 before and 1.8 ± 2.0 after the 6MWT; the mean change was 1.2 ± 1.9. At month 12, the mean Borg scale fatigue scores were 0.7 ± 1.4 before and 1.4 ± 2.1 after the 6MWT; the mean change was 0.7 ± 1.5.

Home Spirometry

In-home spirometry results appeared to remain stable over the LOPAC 12-month follow-up period. Linear model estimation according to treatment in the parent studies showed either a stable or slightly declining average FEV_1; however, the available data on those outcomes were limited because of a high number of missed assessments and a high rate of participant discontinuation.

Continuous Activity and Sleep Monitoring

For participants who consented to continuous monitoring of activity, sleep, and heart rate via a wearable device (e.g., Apple watch), the results generally appeared to remain stable over the LOPAC 12-month follow-up period (Appendix Figure 7). However, data on those outcomes were limited overall because activity monitoring was optional; at month 12, data were only available for 4 participants.

Appendix Table 1. Baseline demographics and clinical characteristics of participants at enrollment into the observational LOPAC study*

Characteristics	Received TCZ in parent study, n = 94	Received other drug† in parent study, n = 41	Received placebo in parent study, n = 38	All participants, N = 173
Age, y, mean (SD)	57.0 (11.3)	56.1 (12.1)	54.7 (13.2)	56.3 (11.9)
Sex				
M	64 (68.1)	24 (58.5)	29 (76.3)	117 (67.6)
F	30 (31.9)	17 (41.5)	9 (23.7)	56 (32.4)
Ethnicity				
Hispanic or Latino	56 (59.6)	19 (46.3)	21 (55.3)	96 (55.5)
Not Hispanic or Latino	38 (40.4)	22 (53.7)	17 (44.7)	77 (44.5)
Race				
White	46 (48.9)	28 (68.3)	17 (44.7)	91 (52.6)
Black or African American	16 (17.0)	2 (4.9)	8 (21.1)	26 (15.0)
Asian	3 (3.2)	3 (7.3)	2 (5.3)	8 (4.6)
American Indian or Alaska Native	2 (2.1)	1 (2.4)	2 (5.3)	5 (2.9)
Native Hawaiian or other Pacific Islander	2 (2.1)	1 (2.4)	1 (2.6)	4 (2.3)
Unknown	25 (26.6)	6 (14.6)	8 (21.1)	39 (22.5)
Tobacco use history				
Never	70 (74.5)	29 (70.7)	25 (65.8)	124 (71.7)
Current	2 (2.1)	0	2 (5.3)	4 (2.3)
Former	22 (23.4)	12 (29.3)	11 (28.9)	45 (26.0)
Medical history‡				
Hypertension	58 (61.7)	29 (70.7)	22 (57.9)	109 (63.0)
Obesity	37 (39.4)	27 (65.9)	17 (44.7)	81 (46.8)
Hyperlipidemia	40 (42.6)	19 (46.3)	17 (44.7)	76 (43.9)
Diabetes	29 (30.9)	17 (41.5)	15 (39.5)	61 (35.3)
Asthma	10 (10.6)	3 (7.3)	6 (15.8)	19 (11.0)
Duration of hospital stay in parent study, d				
Mean (SD)	14.9 (14.2)	13.3 (9.5)	18.9 (17.4)	15.4 (14.1)
Median (range)	10 (3–74)	12 (5–61)	12 (5–72)	11 (3–74)
Time from parent study completion or discontinuation to LOPAC enrollment, d‡				
Mean (SD)	174.4 (90.6)	112.4 (73.5)	180.2 (77.0)	161.0 (87.8)
Median (range)	165 (–1 to 338)	118 (7–278)	159 (26–334)	155 (–1 to 338)
Ordinal scale of clinical status at enrollment in parent study‡				
1, discharged or ready for discharge	0	0	0	0
2, non-ICU hospital ward, not requiring supplemental oxygen	5 (5.3)	1 (2.4)	1 (2.6)	7 (4.0)
3, non-ICU hospital ward, requiring supplemental oxygen	34 (36.2)	10 (24.4)	21 (55.3)	65 (37.6)
4, non-ICU hospital ward, requiring noninvasive ventilation or high-flow oxygen	43 (45.7)	29 (70.7)	14 (36.8)	86 (49.7)
5, ICU, requiring intubation and MV	9 (9.6)	1 (2.4)	2 (5.3)	12 (6.9)
6, ICU, requiring ECMO or MV and additional organ support	3 (3.2)	0	0	3 (1.7)
7, Death	0	0	0	0
Ordinal scale of clinical status at completion of parent study				
1, discharged or ready for discharge	90 (95.7)	40 (97.6)	37 (97.4)	167 (96.5)
2, non-ICU hospital ward, not requiring supplemental oxygen	0	0	0	0
3, non-ICU hospital ward, requiring supplemental oxygen	2 (2.1)	1 (2.4)	1 (2.6)	4 (2.3)
4, non-ICU hospital ward, requiring non-invasive ventilation or high-flow oxygen	1 (1.1)	0	0	1 (0.6)
5, ICU, requiring intubation and MV	1 (1.1)	0	0	1 (0.6)

Characteristics	Received TCZ in parent study, n = 94	Received other drug† in parent study, n = 41	Received placebo in parent study, n = 38	All participants, N = 173
6, ICU, requiring ECMO or MV and additional organ support	0	0	0	0
7, death	0	0	0	0
Supplemental oxygen at enrollment into LOPAC				
Yes	2 (2.1)	0	0	2 (1.2)
Steroid use in parent study‡				
Steroids initiated at parent study baseline	63 (67.0)	26 (63.4)	16 (42.1)	105 (60.7)
Ongoing steroid use in parent study	64 (68.1)	38 (92.7)	28 (73.7)	130 (75.1)
Remdesivir use in parent study‡				
Remdesivir initiated at parent study baseline	18 (19.1)	25 (61.0)	18 (47.4)	61 (35.3)
Ongoing remdesivir use in parent study	13 (13.8)	19 (46.3)	16 (42.1)	48 (27.7)

*Values are no. (%) except as indicated. Demographics (age, sex, ethnicity, race, history of tobacco use) are LOPAC study baseline data; all clinical characteristics are from parent study data. ECMO, extracorporeal membrane oxygenation; ICU, intensive care unit; LOPAC, Long-Term Outcomes Post Acute COVID-19; MV, mechanical ventilation; TCZ, tocilizumab.
†Other drugs include remdesivir, astegolimab, or efmarodocokin alfa or drugs prescribed as part of the standard of care.
‡Values are from the parent study baseline data.

Appendix Table 2. Pulmonary function tests performed at LOPAC study baseline and month 12 measuring lung texture abnormality status*

Tests	LOPAC study, baseline			LOPAC study, month 12		
	≥1 Lung texture abnormality†	No lung texture abnormality	Missing data	≥1 Lung texture abnormality†	No lung texture abnormality	Missing data
FEV_1						
No. participants	85	57	11	53	53	55
Mean, % (SD)	87.8 (19.6)	90.9 (18.1)	NA	92.8 (17.5)	95.2 (15.1)	NA
FVC						
No. participants	85	57	11	53	53	55
Mean, % (SD)	82.7 (17.4)	87.8 (14.8)	NA	87.6 (14.6)	92.5 (16.0)	NA
DLCO corrected for hemoglobin						
No. participants	50	40	11	34	37	55
Mean, % (SD)	79.0 (23.4)	88.8 (16.6)	NA	82.3 (19.8)	94.3 (21.6)	NA

*Values are shown as mean (±SD) percent-predicted values. DLCO, diffusion capacity of the lungs for carbon monoxide; FEV_1, forced expiratory volume in 1 s; FVC, forced vital capacity; HRCT, high-resolution computed tomography; LOPAC, Long-Term Outcomes Post Acute COVID-19; NA, not applicable.
†Defined as ground-glass opacification, reticular pattern, bronchiectasis, hyperlucency, or honeycombing detected on HRCT.

Appendix Table 3. Associations between *MUC5B* promoter risk allele rs35705950 status and lung texture abnormality or lung function impairment*

Lung characteristics	LOPAC study, baseline		LOPAC study, month 12	
	rs35705950 positive, n = 22	rs35705950 negative, n = 149	rs35705950 positive, n = 22	rs35705950 negative, n = 149
Lung texture abnormalities				
Participants with ≥1 lung texture abnormality†	13	77	6	50
Participants with no lung texture abnormalities‡	7	64	6	56
p value§	0.474	NA	>0.999	NA
Lung function impairment¶				
Participants with FVC <80%	10	45	2	24
Participants with FVC ≥80%	10	81	10	73
p value§	0.227	NA	0.727	NA

*FVC, forced vital capacity; LOPAC, Long-Term Outcomes Post Acute COVID-19; NA, not applicable.
†Radiographic evidence of hyperlucency was initially assessed as unknown in 1 participant's baseline and month 12 high-resolution computed tomography scans. This participant was later assessed as having radiographic evidence of hyperlucency at both time points and was included as hyperlucent in the final analysis.
‡Participants were considered to have no lung texture abnormality if there was no radiologic evidence of ground-glass opacification, reticular pattern, bronchiectasis, hyperlucency, or honeycombing on high-resolution computed tomography scans.
§p value calculated by using Fisher exact test.
¶Lung function impairment is defined as <80% of the predicted value.

Appendix Table 4. Pulmonary function tests at LOPAC baseline and month 12 of the LOPAC study according to the parent study treatment cohort*

Tests	Received TCZ in parent study, n = 94	Received other drug† in parent study, n = 41	Received placebo in parent study, n = 38	All participants, N = 173
FEV_1 at LOPAC baseline				
No. participants	75	40	32	147
Mean, % (SD)	88.0 (21.1)	86.2 (14.3)	89.4 (23.2)	87.8 (19.9)
Median, % (range)	91.1 (29–145)	84.5 (65–119)	89.9 (29–138)	89.0 (29–145)
FEV_1 at month 12				
No. participants	52	30	27	109
Mean, % (SD)	97.6 (17.8)	88.6 (14.9)	92.0 (12.6)	93.7 (16.2)
Median, % (range)	97.9 (34–134)	86.5 (68–120)	91.0 (70–121)	93.0 (34–134)
Change in FEV_1 from baseline to month 12				
No. participants	43	30	23	96
Mean, % (SD)	2.0 (10.8)	0.7 (7.5)	2.5 (12.3)	1.7 (10.3)
Median, % (range)	2.1 (−26 to 20)	1.6 (−25 to 16)	3.0 (−31 to 27)	2.9 (−31 to 27)
FVC at LOPAC baseline				
No. participants	75	40	32	147
Mean, % (SD)	85.9 (18.4)	82.0 (13.7)	84.5 (15.5)	84.5 (16.6)
Median, % (range)	88.3 (33–126)	82.1 (56–112)	83.9 (48–120)	84.9 (33–126)
FVC at month 12				
No. participants	52	30	27	109
Mean, % (SD)	94.6 (16.6)	84.7 (14.7)	86.6 (10.3)	89.9 (15.3)
Median, % (range)	94.0 (41–142)	83.5 (65–115)	87.8 (67–111)	89.0 (41–142)
Change in FVC from baseline to month 12				
No. participants	43	30	23	96
Mean, % (SD)	2.4 (10.1)	1.8 (7.4)	4.9 (9.1)	2.8 (9.1)
Median, % (range)	3.0 (–21 to 23)	2.5 (–22 to 23)	3.5 (–14 to 28)	3.0 (–22 to 28)

*Data for FEV_1 and FVC are shown as percent-predicted values. FEV_1, forced expiratory volume in 1 s; FVC, forced vital capacity; LOPAC, Long-Term Outcomes Post Acute COVID-19; TCZ, tocilizumab.

†Other drugs include study treatment with remdesivir, astegolimab, or efmarodocokin alfa or drugs prescribed as part of the standard of care.

Appendix Table 5. Proportion of participants with lung texture abnormalities on HRCT at LOPAC baseline and Month 12 by parent study treatment cohort*

	No. patients (%)			
HRCT lung characteristics	Received TCZ in parent study, n = 94	Received other drug† in parent study, n = 41	Received placebo in parent study, n = 38	All participants, N = 173
At LOPAC study baseline				
Participants with ≥1 lung texture abnormality	49 (52.1)	25 (61.0)	17 (44.7)	91 (52.6)
Ground-glass opacification	46 (48.9)	23 (56.1)	17 (44.7)	86 (49.7)
Reticular pattern	7 (7.4)	5 (12.2)	3 (7.9)	15 (8.7)
Bronchiectasis	5 (5.3)	3 (7.3)	4 (10.5)	12 (6.9)
Hyperlucency‡	2 (2.1)	0	0	2 (1.2)
Honeycombing	0	0	0	0
Participants with no lung texture abnormality§	37 (39.4)	16 (39.0)	18 (47.4)	71 (41.0)
Participants with missing data	8 (8.5)	0	3 (7.9)	11 (6.4)
At month 12 of LOPAC study				
Participants with ≥1 lung texture abnormality	28 (29.8)	15 (36.6)	13 (34.2)	56 (32.4)
Ground-glass opacification	24 (25.5)	14 (34.1)	13 (34.2)	51 (29.5)
Reticular pattern	4 (4.3)	4 (9.8)	2 (5.3)	10 (5.8)
Bronchiectasis	2 (2.1)	2 (4.9)	3 (7.9)	7 (4.0)
Hyperlucency‡	2 (2.1)	0	0	2 (1.2)
Honeycombing	0	0	0	0
Participants with no lung texture abnormality§	32 (34.0)	15 (36.6)	15 (39.5)	62 (35.8)
Participants with missing data	34 (36.2)	11 (26.8)	10 (26.3)	55 (31.8)

*HRCT, high-resolution computed tomography; LOPAC, Long-Term Outcomes Post Acute COVID-19; TCZ, tocilizumab.

†Other drugs include remdesivir, astegolimab, or efmarodocokin alfa or drugs prescribed as part of the standard of care.

‡ Radiographic evidence of hyperlucency was assessed as unknown in 1 participant's baseline and HRCT images. This participant was considered to have radiographic evidence of hyperlucency at baseline and month 12.

§Participants were considered to have no lung texture abnormality if there was no radiologic evidence of ground-glass opacification, reticular pattern, bronchiectasis, hyperlucency, or honeycombing on HRCT scan.

Appendix Table 6. MoCA scores indicating cognitive function at baseline and month 12 of the LOPAC study according to parent study treatment cohort*

MoCA scores†	Received TCZ in parent study, n = 94	Received other drug‡ in parent study, n = 41	Received placebo in parent study, n = 38	All participants, N = 173
At LOPAC baseline				
No. participants	90	39	36	165
Mean (SD)	23.9 (4.0)	24.9 (3.7)	25.1 (3.7)	24.4 (3.9)
Median (range)	25.0 (9–30)	26.0 (17–30)	26.0 (16–30)	25.0 (9–30)
At month 12				
No. participants	55	30	28	113
Mean (SD)	24.6 (4.2)	25.7 (3.1)	25.1 (4.1)	25.0 (3.9)
Median (range)	25.0 (12–30)	27.0 (16–30)	26.0 (14–30)	26.0 (12–30)
Change in MoCA score from baseline to month 12				
No. participants	55	30	28	113
Mean change (SD)	1.0 (3.2)	0.8 (3.3)	−0.0 (2.8)	0.7 (3.1)
Median change (range)	1.0 (−9 to 8)	1.0 (−5 to 10)	0.0 (−6 to 5)	1.0 (−9 to 10)

* LOPAC, Long-Term Outcomes Post Acute COVID-19; MoCA, Montreal Cognitive Assessment; TCZ, tocilizumab.
†The lower the MoCA score, the greater the cognitive impairment.
‡Other drugs include remdesivir, astegolimab, or efmarodocokin alfa, or drugs prescribed as part of the standard of care.

Appendix Table 7. Vitality scores from the SF-36v2 questionnaire at baseline and month 12 of the LOPAC study according to parent study treatment cohort*

Vitality score	Received TCZ in parent study, n = 94	Received other drug† in parent study, n = 41	Received placebo in parent study, n = 38	All participants, (N = 173
At LOPAC study baseline				
No. participants	87	40	37	164
Mean (SD)	60.9 (22.6)	59.3 (20.0)	59.1 (21.7)	60.1 (21.7)
Median (range)	62.5 (13–100)	59.4 (0–94)	62.5 (13–100)	62.5 (0–100)
At month 12				
No. participants	58	30	29	117
Mean (SD)	69.3 (19.7)	68.8 (17.5)	68.8 (21.3)	69.0 (19.4)
Median (range)	68.8 (19–100)	68.8 (31–100)	75.0 (13–100)	68.8 (13–100)
Change in vitality score from baseline to month 12				
No. participants	58	30	29	117
Mean (SD)	7.9 (22.3)	6.8 (19.5)	10.1 (17.3)	8.2 (20.3)
Median (range)	6.3 (−50 to 63)	6.3 (−25 to 44)	6.3 (−19 to 44)	6.3 (−50 to 63)

*Vitality was assessed by using the SF-36v2 questionnaire. Normal was defined as a score ≥50 points (range 0–100 points) for the vitality domain. LOPAC, Long-Term Outcomes Post Acute COVID-19; SF-36v2, Short Form 36 Question Health Survey version 2; TCZ, tocilizumab.
†Other drugs include remdesivir, astegolimab, or efmarodocokin alfa, or drugs prescribed as part of the standard of care.

Appendix Table 8. Aggregated healthcare resource utilization throughout the 12-month follow-up period or the LOPAC study*

Resource Utilization	LOPAC study participants, no. (%), N = 173†
Telephone health advice	61 (35.3)
Attendance at the hospital as an outpatient	57 (32.9)
Emergency department visit(s)	43 (24.9)
Physical therapy	35 (20.2)
Urgent care/unscheduled visit(s)	31 (17.9)
Hospitalization	19 (11.0)
Nurse home visits	17 (9.8)
Rehabilitation	15 (8.7)
Physician home visits	3 (1.7)
Admittance to ICU during hospitalization	2 (1.2)
Other‡	70 (40.5)

*ICU, intensive care unit; LOPAC, Long-Term Outcomes Post Acute COVID-19.
†A total of 129 participants completed the questionnaire at least once during the study.
‡Most common healthcare resource utilization categorized as other was primary care visits.

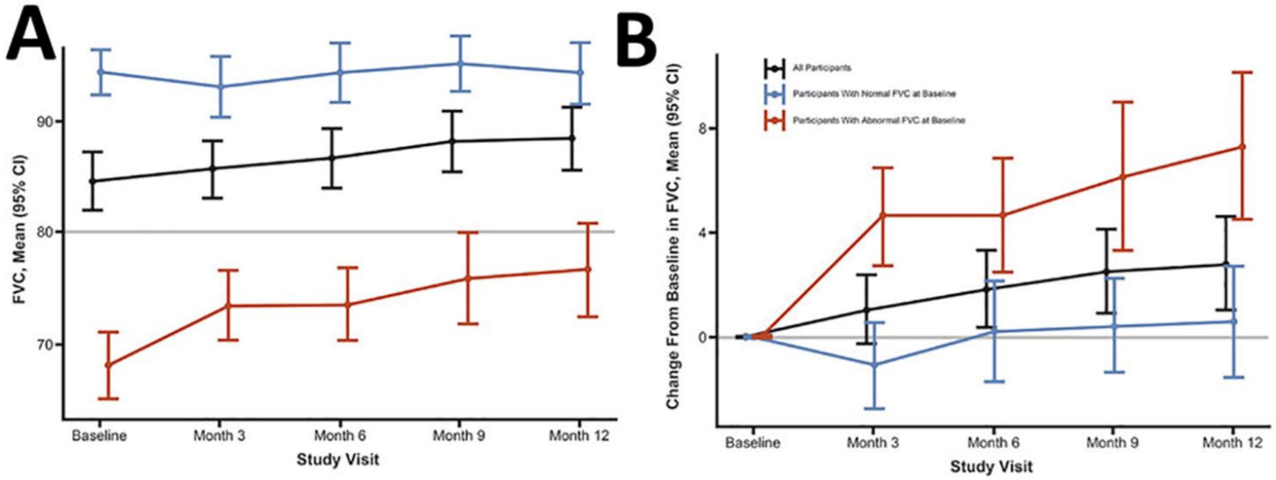

Appendix Figure 1. Longitudinal FVC profiles for participants of the Long-Term Outcomes Post Acute COVID-19 study. Data are shown as mean (95% CI) percent-predicted values. A) Mean percent-predicted FVC at different study visits. B) Mean change of percent-predicted FVC during study visits according to the study baseline. Normal is defined as >80% of predicted value. The gray horizonal lines show 80% predicted FVC (A) and no change in percent-predicted FVC (B). FVC, forced vital capacity.

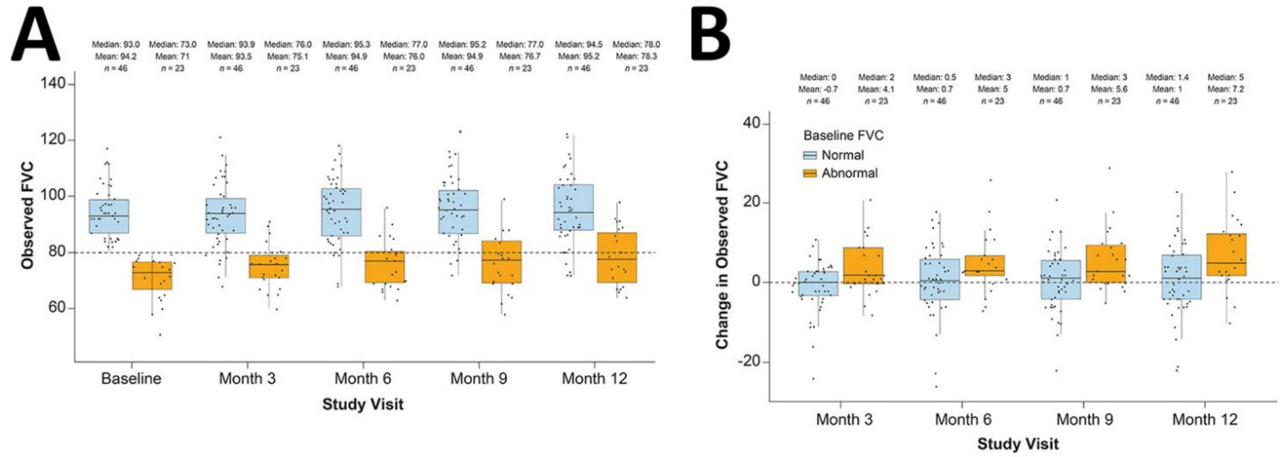

Appendix Figure 2. Longitudinal profiles of observed FVC (A) and change from baseline (B) according to FVC status at baseline among participants who completed all visits during the 12-month Long-Term Outcomes Post Acute COVID-19 study. Horizontal lines within boxes indicate medians; box tops and bottoms indicate upper (third) and lower (first) quartiles; error bars (whiskers) indicate 1.5 × interquartile range. Normal is defined as >80% predicted value; abnormal is defined as <80%. Dashed horizontal line in (A) at 80% represents the cutoff value for abnormal FVC. FVC, forced vital capacity.

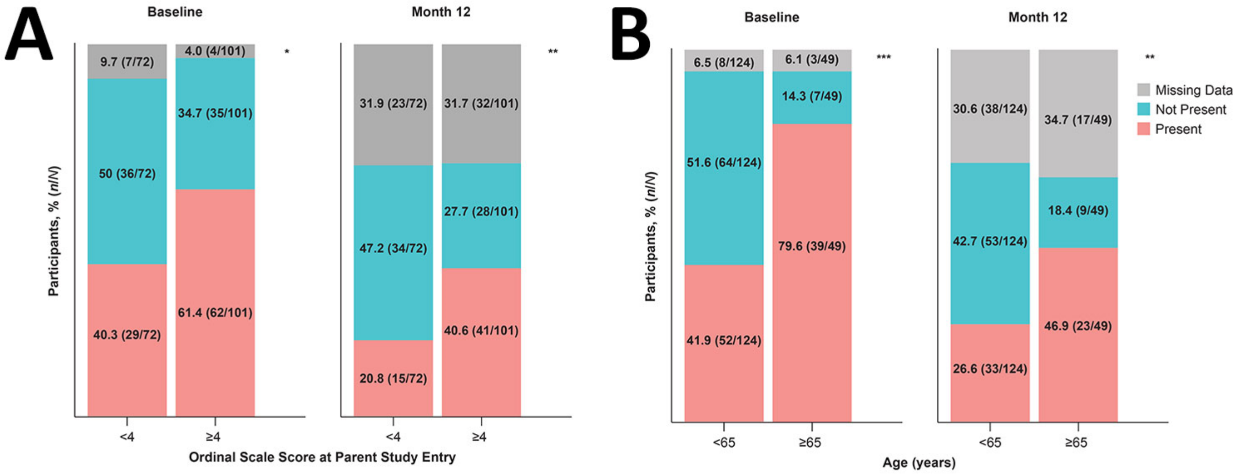

Appendix Figure 3. Percentages of participants with >1 lung texture abnormality on high-resolution computed tomography at baseline and month 12 according to ordinal scale score at parent study baseline (A) and age group (B). *, p<0.05; **, p<0.01; ***, p<0.001.

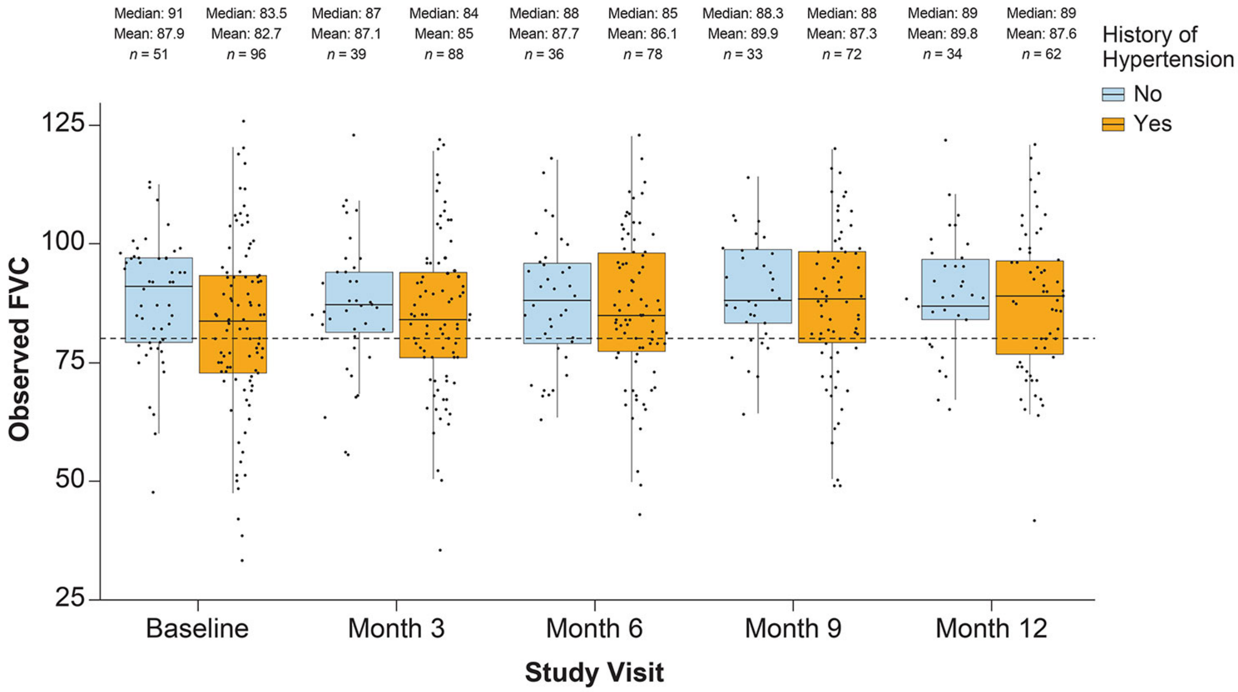

Appendix Figure 4. Box plots of longitudinal profiles of observed FVC according to history of hypertension. Horizontal lines within boxes indicate medians; box tops and bottoms indicate upper (third) and lower (first) quartiles; error bars (whiskers) indicate 1.5 × interquartile range. Dashed horizontal line at 80% represents the cutoff value for abnormal FVC (<80% of the predicted value was defined as abnormal). FVC, forced vital capacity.

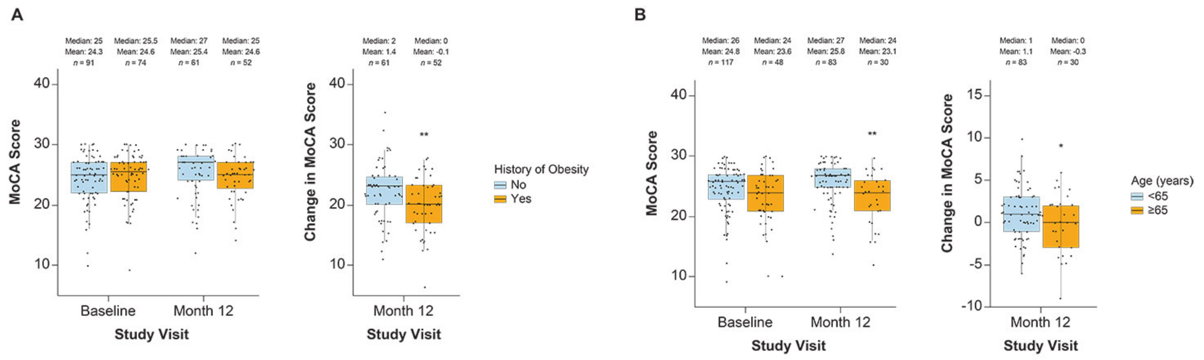

Appendix Figure 5. Longitudinal profiles of MoCA scores and change from baseline at month 12 according to obesity (A) and age (B) in the Long-Term Outcomes Post Acute COVID-19 study. Horizontal lines within boxes indicate medians; box tops and bottoms indicate upper (third) and lower (first) quartiles; error bars (whiskers) indicate 1.5 × interquartile range. Data points beyond the end of the whiskers are considered outliers. MoCA, Montreal Cognitive Assessment. *, p<0.05; **, p<0.01.

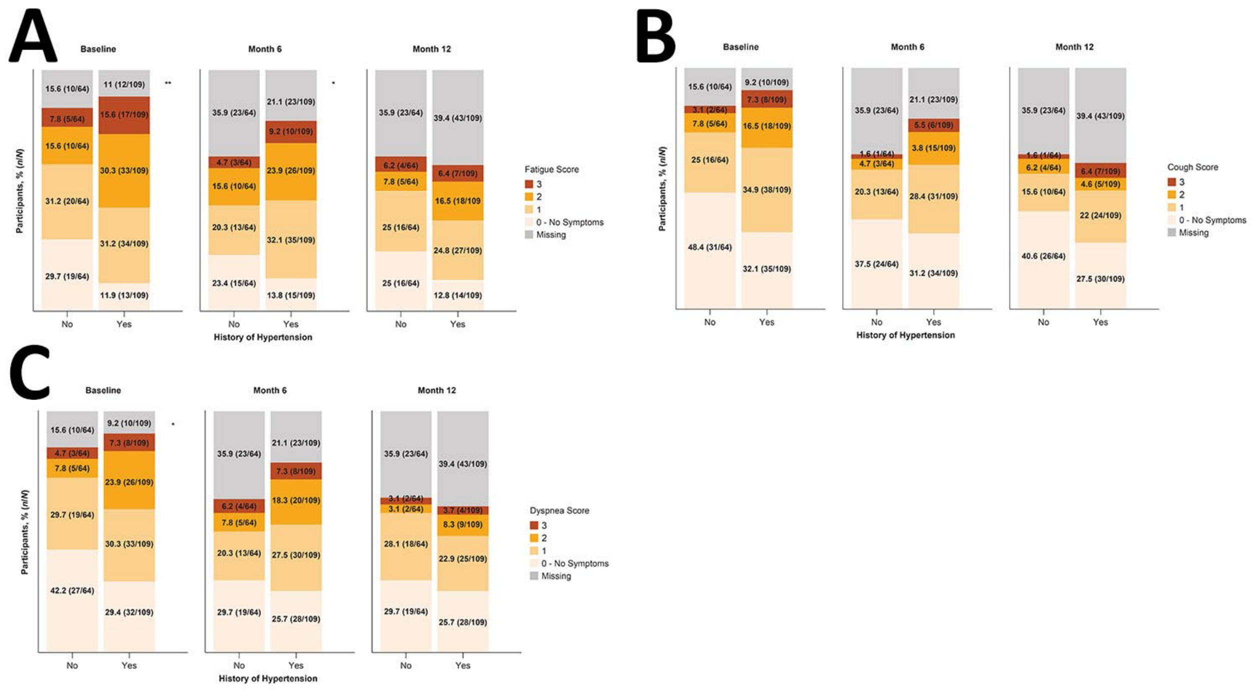

Appendix Figure 6. Longitudinal profiles of patient-reported symptoms from the Modified Living with Idiopathic Pulmonary Fibrosis questionnaire assessment according to history of hypertension. Symptoms were fatigue (A), cough (B), and dyspnea (C). χ^2 test of independence was used to test the association between a history of hypertension and a symptom score of 0 versus >0. Scores ranged from 0 to 3; the lowest score corresponds to no symptoms and the higher score corresponds to greater symptom severity. *, p<0.05; **, p<0.01.

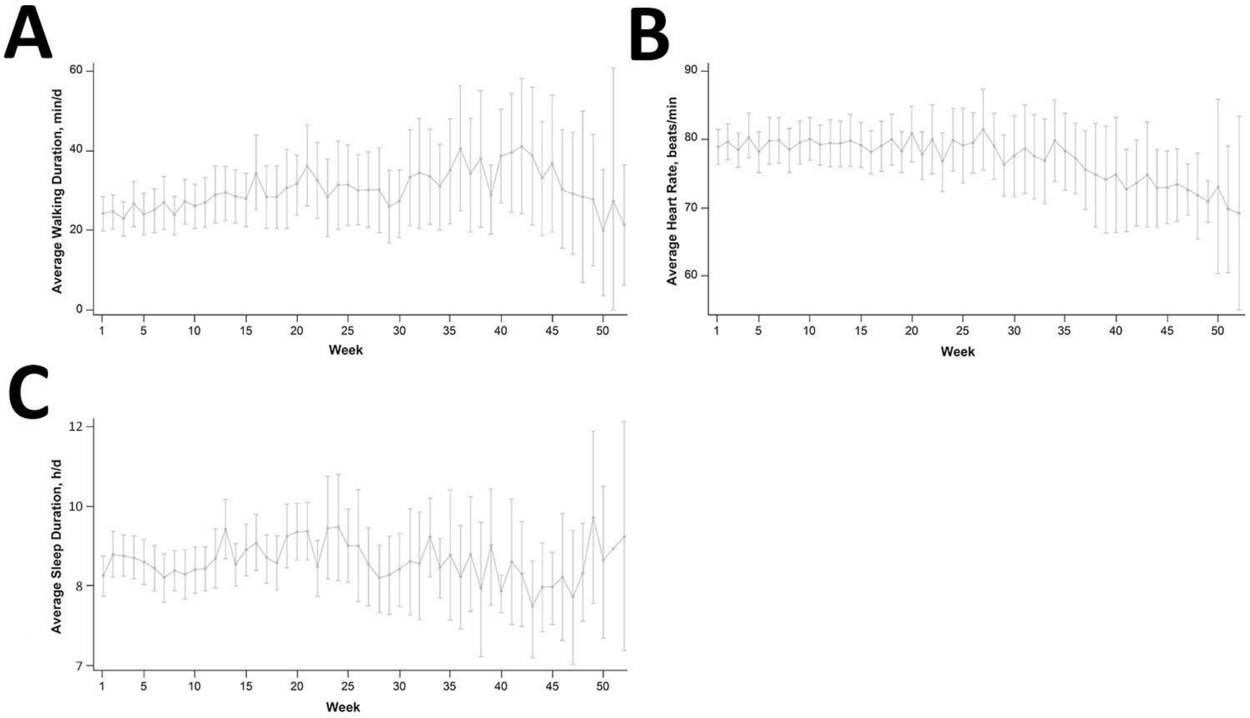

Appendix Figure 7. Continuous monitoring of activity, heart rate, and sleep from baseline to month 12 during the Long-Term Outcomes Post Acute COVID-19 study. Weekly average walking duration (A), heart rates (B), and sleep duration (C) in study participants.
